# Centralising acute stroke care within clinical practice in the Netherlands: lower bounds of the causal impact

**DOI:** 10.1186/s12913-020-4959-3

**Published:** 2020-02-10

**Authors:** Roel D. Freriks, Jochen O. Mierau, Erik Buskens, Elena Pizzo, Gert-Jan Luijckx, Durk-Jouke van der Zee, Maarten M. H. Lahr

**Affiliations:** 1 Department of Economics, Econometrics & Finance, Faculty of Economics & Business, University of Groningen, Groningen, The Netherlands; 2 Unit Patient Centred Health Technology Assessment, Department of Epidemiology, University of Groningen, University Medical Centre Groningen, Groningen, The Netherlands; 3 Aletta Jacobs School of Public Health, Groningen, The Netherlands; 4 Department of Operations, Faculty of Economics & Business, University of Groningen, Groningen, The Netherlands; 5 Department of Applied Health Research, Faculty of Population Health Sciences, University College London, London, England; 6 Department of Neurology, University of Groningen, University Medical Centre Groningen, Groningen, The Netherlands

**Keywords:** Acute stroke care, Organisational system, Evaluation, Observational data

## Abstract

**Background:**

Authors in previous studies demonstrated that centralising acute stroke care is associated with an increased chance of timely Intra-Venous Thrombolysis (IVT) and lower costs compared to care at community hospitals. In this study we estimated the lower bound of the causal impact of centralising IVT on health and cost outcomes within clinical practice in the Northern Netherlands.

**Methods:**

We used observational data from 267 and 780 patients in a centralised and decentralised system, respectively. The original dataset was linked to the hospital information systems. Literature on healthcare costs and Quality of Life (QoL) values up to 3 months post-stroke was searched to complete the input. We used Synthetic Control Methods (SCM) to counter selection bias. Differences in SCM outcomes included 95% Confidence Intervals (CI). To deal with unobserved heterogeneity we focused on recently developed methods to obtain the lower bounds of the causal impact.

**Results:**

Using SCM to assess centralising acute stroke 3 months post-stroke revealed healthcare savings of $US 1735 (CI, 505 to 2966) while gaining 0.03 (CI, − 0.01 to 0.73) QoL per patient. The corresponding lower bounds of the causal impact are $US 1581 and 0.01. The dominant effect remained stable in the deterministic sensitivity analyses with $US 1360 (CI, 476 to 2244) as the most conservative estimate.

**Conclusions:**

In this study we showed that a centralised system for acute stroke care appeared both cost-saving and yielded better health outcomes. The results are highly relevant for policy makers, as this is the first study to address the issues of selection and unobserved heterogeneity in the evaluation of centralising acute stroke care, hence presenting causal estimates for budget decisions.

## Background

The care that patients receive following hospital discharge largely determined the high costs of stroke [1, 2]. Treatment with Intra-Venous Thrombolysis (IVT) is cost-effective as the health benefits outweighed the initial costs [3, 4]. Although IVT treatment rates have risen in the last decade [5], there is still substantial undertreatment given the fact that currently between 8 and 10% of patients were treated [6, 7], whereas treatment rates up to 30% have been achieved in optimised and dedicated settings [8]. There are various reasons for the current undertreatment of patients with IVT. These can largely be grouped in themes such as patient delay, performance of the stroke pathway and the organisational system in place for providing acute care [8].

Centralising care in designated stroke centres resulted in more patients arriving in time for treatment, improved outcomes and lowered mortality rates compared to care provided in community hospitals [9–13]. Potential factors influencing more timely hospital arrival of patients within centralised settings were a lower threshold for using ambulance services and preferential routing of patients with suspected stroke candidate for acute treatment [14]. Also a greater awareness and readiness for IVT may exist among healthcare professionals in a centralised organisational system [9]. This can be the result of a combination of experience and exposure to IVT, continued medical training and new trainees entering the workforce [15, 16]. Improvement in outcome is based on a larger proportion of patients arriving on time for treatment at the hospital and a shorter time to treatment (door-to-needle time) within the hospital [9, 17]. In the Northern Netherlands a centralised organisational system for acute stroke care was developed in which patients with suspected stroke are transported to a single tertiary university hospital for acute treatment [9]. We have learned from previous research that a centralised system can be associated with a 50% increased chance of treatment compared to a decentralised system in which treatment is offered in community hospitals.

Using a probabilistic simulation modelling, a recent study showed that centralising IVT would substantially lower mean annual costs per patient compared to improving care at community hospitals separately [17]. However, the causal impact of centralising acute stroke care within clinical practice remained unclear. There is previously demonstrated that centralising stroke care systems was cost-effective, improved outcomes and reduced mortality and costs [3, 18, 19]. Yet, these studies did not adequately counter the endogeneity in the comparison, which limited a causal interpretation of the delivered estimates. Specifically, both selection into centralised stroke care systems and the inference on assessed outcomes are potentially driven by other factors. Hence, not taking these (un) observables into account may have yielded biased estimates, possibly resulting in suboptimal policy decisions. In this study we specifically link this omitted variable bias to the coefficient stability, enabling identification of the lower bound of impact on cost and health outcomes 3 months post-stroke.

## Methods

### Stroke system characteristics

In the Northern Netherlands, a centralised and decentralised stroke care system for acute stroke care co-exist [9]. Within the centralised system acute stroke treatment is performed in the University Medical Centre Groningen (UMCG), a tertiary university hospital. Within the catchment area of four hospitals, arrangements were made with hospitals, General Practitioners (GPs) and Emergency Medical Services (EMS) to bypass the local three community hospitals, and transfer potential stroke victims directly to the UMCG for acute stroke treatment. Approximately 580.000 inhabitants are served by the centralised system, with a population density of 250 inhabitants per square kilometer. The decentralised system consists of nine community hospitals all offering IVT to patients with suspected acute stroke in their catchment area. Both stroke care systems conform to the national guidelines. All hospitals practice identical protocols for identification of patients with suspected stroke, triage and 911 systems, ambulance transport and finally IVT treatment.. For the patients within the centralised system this meant possibly bypassing a community hospital and being taken to a comprehensive stroke center directly. A total of 1.14 million inhabitants are served by the decentralised system, with an average population density of 189 inhabitants per square kilometer. For the whole of the Northern Netherlands, geography is quite similar with low levels of traffic congestion, the absence of mountains and a temperate maritime climate.

### Data sources

We used patient-level data from 1047 stroke patients who were part of a large observational study carried out in the Northern Netherlands in 2010 over the course of 6 months [9]. Of these patients, 780 patients were admitted to community hospitals all part of a decentralised stroke care system, and 267 patients were admitted to a centralised stroke care system. The descriptive statistics of the patients are presented in Table 1. Within the centralised system ischemic stroke patients from all four hospitals were considered. The original dataset was linked to the hospital information systems to acquire additional information for the calculation of hospital costs, such as length of stay. A description of the number of stroke presentations at each included hospital is provided in Table 6 in Appendix.

**Table 1:** Descriptive statistics

	Centralised	Decentralised
Number of patients	267	780
Age in years (SD)	70 (14)	73 (13)^a^
Male (%)	149 (56)	383 (49)^a^
IVT received (%)	61 (23)^b^	112 (14)
Median sNIHSS on arrival (IQR)	1 (0–3)	1 (0–3)
Median mRS at 3 months (IQR)	1 (0–5)	2 (0–5)^a^
Referral GP (%)	101 (38)	437 (56)^a^
First responder EMS (%)	78 (29)	178 (23)
Transported by EMS (%)	204 (76)	456 (58)^b^
Median distance to hospital (km)	15.6	9.3^b^

SD indicates standard deviation; *IVT* Intravenous Thrombolysis, *sNIHSS* short National Institutes of Health Stroke Scale, *IQR* Interquartile Range, *mRS* modified Rankin Scale, *GP* General Practitioner, *EMS* Emergency Medical Services, *km* kilometer. Inference: ^a^/^b^ indicate significant differences at the 5%/1% level based on the mean differences of the two systems

### Approach

We used patient-level data from a previously published study on a central and decentral stroke care system in the Northern Netherlands [9]. Costs from onset to treatment had been collected in prior work [17] and extended by linking the original dataset [9] to the hospital information system to include intra-hospital costs. The Costs after hospital discharge up to 3 months were based on the literature [20]. Functional disability and independence at 3 months was assessed with the modified Rankin Scale (mRS). mRS scores were subsequently mapped into Quality of Life (QoL) values using a validated algorithm [21, 22].

### Health measures

#### Short National Institutes of Health stroke scale (sNIHSS)

The sNIHSS is a commonly used scale to measure stroke severity in the pre-hospital phase, but has also been used in hospital settings [23]. We used the 5-item sNIHSS, covering gaze, visual fields, motor function in both legs and language. The sNIHSS scores were recorded in the original dataset and used as a measure for patients’ health upon hospital arrival.

#### Quality of life (QoL) values

The mRS score is a commonly used scale to measure disability and independence in stroke victims [24]. The scale consists of seven grades, from 0 to 6, with 0 corresponding to no symptoms, 5 corresponding to severe disability and 6 to indicate mortality. The mRS scores at 3 months were recorded in the original dataset and mapped into QoL values between 0 and 1 using a validated algorithm [21], implemented with the corresponding STATA package mrs2eq [22].

The EQ. 5D questionnaire is a standardized instrument developed by the EuroQol Group as a measure of QoL that can be used in a wide range of health conditions and treatments [25]. The QoL values were used as a one-time measure for patients’ health at 3 months post stroke. Pre-stroke QoL values were missing, making the calculation of Qualtiy-Adjusted Life Years (QALYs) not straightforward as information on time spent within the first 3 months is missing.

### Cost calculation

The health care use of both systems was ascertained and valued. Unit costs were obtained from the Dutch Manual of Costing [26]. The costs associated with healthcare use are presented in Table 2. The original dataset [9] was linked to the hospital information systems to collect the intra-hospital costs. Data linkage with the hospital information system, PoliPlus, was requested by the researchers and performed by hospital’s neurology department. All patients in the original dataset [9] were linked with the system. Costs in the post-hospital phase were based on cost estimates previously published in a Dutch setting [21] combined with the observed destination and functional independence at hospital discharge. Costs were determined from a healthcare provider perspective. Productivity losses due to functional impairments were not considered, since the average age of the sample is above retirement age and relevant measures for the sample below retirement age were not available in the dataset.

**Table 2:** Unit costs associated with healthcare use

Resource	Unit costs ($US)	Source
Variable costs		
General practitioner		(1)
Telephonic consultation	$19.04	
Visit by general practitioner	$56.00	
Emergency medical services transport		(2)
Emergency transport	$882.00	
Dispatch	$71.00	
Per driven kilometer	$5.00	
Medical personnel ER visit		(1)
Medical specialist (15 min)	$44.38	
Resident (1 h)	$36.48	
Nurse (1 h)	$35.04	
Outpatient clinic visit	$89.60	(1)
Computed tomography scan	$144.48	(3)
Central laboratory (per test)	$27.10	(4)
Alteplase	$532.46	(5)
Neurology ward (1 day)	$466.10	(1)
Stroke unit (1 day)	$626.68	(3)
Care after discharge		(1)
Home care (1 day)	$59.00	
Remedial therapy (1 session)	$38.94	
Rehabilitation centre (1 day)	$542.80	
Nursing home (1 day)	$198.24	

$US indicates United States dollar; ER, emergency room. (1) Dutch manual of costing [26]; (2) Data from regional ambulance services Groningen; (3) Dirks et al., 2012 [20]; (4) Claes et al., 2006 [27]; (5) www.medicijnkosten.nl [28]

#### Pre-hospital costs

Pre-hospital costs were based on mode of referral (GP, 911, self-referral, or intra-hospital), ambulance transportation and distance covered by EMS [17]. The indicators were multiplied with the unit prices as presented in Table 2.

#### Intra-hospital costs

Intra-hospital costs were based on whether the patient was treated with IVT, length-of-stay in the acute stroke unit and length-of-stay in the neurology ward. For this, the original dataset was linked to the hospital information system which contains detailed medical information on length of stay at the neurology department. Differences in staffing costs between university medical centres and community hospitals were taken into account [26].

#### Costs after hospital discharge

Costs after hospital discharge up to 3 months were not directly observed. We adopted the strategy of Dirks et al. [20] and related mRS scores at 3 months to average healthcare use after discharge. Patients in the mRS 0–1 category were presumed to be discharged home with no extra costs. Patients in the mRS 2–3 category were presumed to be discharged home with additional home care (1 h/day) and remedial therapy costs (3 sessions/week). Patients in the mRS 4 category were discharged (depending on age) to a rehabilitation centre (if younger than 65 years) or a nursing home (if aged 65 years or older). Patients in the mRS 5 category were discharged to a nursing home. mRS 6 category means deceased with no extra costs.

#### Adjustment for timing and currency

The index year is 2019. Therefore, costs are corrected with an average annual inflation rate of 1.015% [29]. Furthermore, since costs were collected from a healthcare provider perspective, cost prices are converted using the current Purchasing Power Parity (PPP) of 1.2642$US per 1 Euro [30].

### Statistical analysis

Mean differences in the patients’ characteristics, costs and health outcomes were determined with independent samples t-tests (normal distribution) or Mann-Whitney U tests (skewed distribution). Mean differences tests on the cost and health outcomes indicated that mean regressions could be used for the estimation.

The regression formulation of the evaluation in this study is given by

1 $$ {Y}_i={c}_0+\beta \times {CS}_i+\boldsymbol{\gamma} \times {\boldsymbol{X}}_i+{e}_i, $$

where *Y*_*i*_ is the outcome of interest (cost, health) for individual *i*, *c*_0_ the intercept, *CS*_*i*_ is a binary variable for the stroke care system with the centralised stroke system as reference category with *β* as the corresponding coefficient, ***X***_*i*_ are the control variables gender, age, IVT received, mode of referral, stroke severity on arrival, and transported by EMS with ***γ*** as the vector of corresponding coefficients and *e*_*i*_ denotes the error term. Distance to hospital was excluded as control variable due to collinearity with the system indicator variable *CS*_*i*_. As mentioned above, Ordinary Least Squares (OLS) regression of eq. (1) yields a biased estimate of *β*, as both selection into centralised stroke care systems and the inference on assessed outcomes are potentially driven by other factors, i.e. *E*[*Y*_*i*_| *e*_*i*_] ≠ 0.

To counter selection bias we use Synthetic Control Methods (SCM) and estimate eq. (1) in two stages. In the first stage we estimate the individual propensity scores of selection in a centralised stroke care system conditional on the control variables ***X***_*i*_ with a logit model denoted by

2 $$ {p}_i=\Pr \left[C{S}_i=1\right|{\boldsymbol{X}}_i\Big] $$

where we followed Rosenbaum and Rubin (1985) and used a preset caliper size of a quarter of a standard deviation of the logit of the propensity score [31, 32]. Mean differences of the raw and matched data and balance plots were used to assess the balancing assumption in the first stage. Subsequently, in the second stage we use the predicted values *p*_*i*_ of eq. (2) to obtain the Average Treatment Effect (ATE),

3 $$ E\left({Y}_i\right|C{S}_i=1,{\boldsymbol{X}}_i\left)-E\left({Y}_i\right|C{S}_i=0,{\boldsymbol{X}}_i\right) $$

The SCM does not control for unobserved heterogeneity, i.e. factors related to the inference on *β* that were not observed in the dataset (e.g., socioeconomic status). Therefore, to assess to what extent the inference on coefficient *β* in eq. (1) is affected by (un) observables we link the omitted variable bias to the coefficient stability using the Altonji ratio [33, 34]. Subsequently, we implement a recently published estimator [35] to obtain the lower bound of the causal effect of centralising acute stroke care denoted by

4 $$ {\hat{\beta}}_{\ast }={\hat{\beta}}_F-\left({\hat{\beta}}_R-{\hat{\beta}}_F\right)\times \frac{R_{\mathit{\operatorname{MAX}}-{R}_F}}{R_F-{R}_R}, $$

where *R*_*F*_ (*R*_*R*_) and ${\hat{\beta}}_F$ (${\hat{\beta}}_R$) are the R-squared and obtained estimate of OLS regression on the full (restricted) model of equation (1), respectively, and *R*_*MAX*_ is the maximum R-squared. The calculation of *R*_*MAX*_ is pre-determined. For example, Bellows & Miquel (2009) suggest *R*_*MAX*_ equals *R*_*F*_ + (*R*_*F*_ − *R*_*R*_) [36]. For that case, Angelini & Mierau (2018) show that ${\hat{\beta}}_{\ast }$ then reduces to $2{\hat{\beta}}_F-{\hat{\beta}}_R$, which is a straightforward way to assess ${\hat{\beta}}_{\ast }$ without further knowledge of the underlying R-squared [37]. Alternatively, Oster (2017) suggest *R*_*MAX*_ equals 1.3 × *R*_*F*_ [35], determined from published randomized controlled trials in leading economic journals between 2008 and 2013. We adopted the latter option, as it incorporates both the coefficient movement and the model’s fit.

Deterministic sensitivity analyses were undertaken to test the stability of the observed estimates. First, we focused on the mapping method of the QoL values. In the sensitivity analysis we used the second validated algorithm of Rivero-Arias et al. (2010) [21] and replicated the OLS regression option using Monte Carlo simulation with 10,000 iterations, again implemented with the STATA package mrs2eq [22]. Second, we focused on the uncertainty underlying the cost derivation of costs after hospital discharge, as this part is largely determined from previously published cost estimates for the Dutch setting [20]. Specifically, we modified the assumptions in the main analysis and presumed that patients in the mRS 4 category either go home during the weekends or receive informal care half a week.

Differences in outcomes include 95% Confidence Intervals (CI). All of above statistical analyses were performed with STATA/SE 15.0 (STATA; https://www.stata.com/).

## Results

### Comparing stroke care systems

A summary on patient recruitment, baseline patient characteristics, access to healthcare services and health outcomes of both stroke care systems is provided in Table 1. Mean differences were determined with independent samples t-tests (normal distribution) or Mann-Whitney U tests (skewed distribution). We observed that while stroke severity on arrival does not differ between the two systems (*P* = 0.132), at 3 months after hospital discharge the level of disability and dependence is greater in the decentralised system than in the centralised system (*P* = 0.012).

In Table 3 the cost composition of both systems is provided. We observed that while the mean pre-hospital costs were greater for the centralised system (*P* = 0.000), the total costs up to 3 months were less than for the decentralised system (*P* = 0.009).

**Table 3:** Cost composition ($US)

	Centralised	Decentralised
Mean pre-hospital costs (CI)	1023** (954–1092)	760 (715–805)
Mean intra-hospital costs (CI)	3722** (3611 – 3832)	3920 (3854 – 3986)
Mean costs after hospital discharge (CI)	3605** (2630 – 4580)	5232 (4669 – 5795)
Mean total costs (CI)	8332** (7271 – 9394)	9944 (9317 – 10,571)

Inference: ** indicate significant differences at the 1% level based on the mean differences of the two systems

### Estimation results

#### Synthetic control methods

**Fig. 1:**
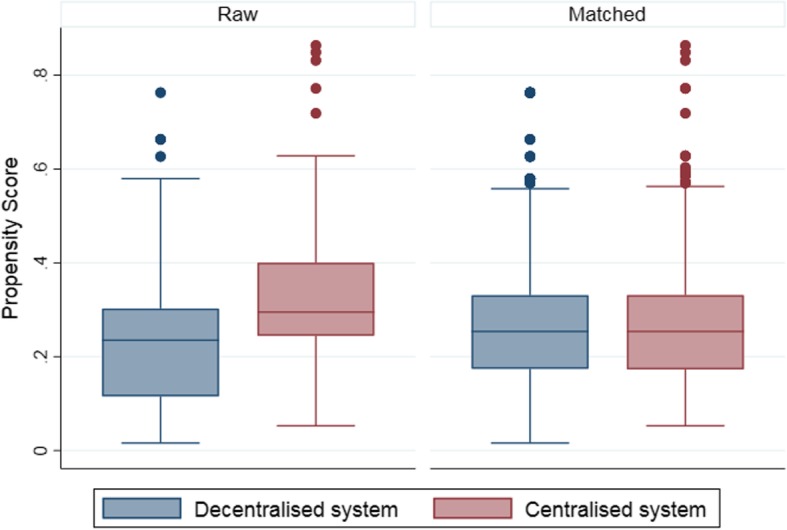
Balance plot of raw and matched data

As mentioned above, we followed Rosenbaum and Rubin (1985) and used a preset caliper size of a quarter of a standard deviation of the logit of the propensity score [31, 32]. No observations were excluded. The systems were balanced in the first stage on the included covariates, as demonstrated with mean differences of the raw and matched data in Table 7 in Appendix and illustrated in the balance plot in Figure 1 in Appendix. The balancing assumption enables to estimate the ATE in the second stage. Using SCM we obtain a $$ \hat{\beta} $$ for healthcare savings and QoL gain of $US 1735 (CI, 505 to 2966) (*P* = 0.006) and 0.03 (CI, − 0.01 to 0.73) (*P* = 0.093), respectively.

#### Causal approach

In Tables 4 and 5 we present the restricted and full coefficients for *β* in equation (1) for incremental healthcare costs and QoL values, respectively. Using $$ {\hat{\beta}}_R $$ and $$ {\hat{\beta}}_F $$ in the first row in combination with *R*_*R*_ and *R*_*F*_ in the last row enables to determine the lower bounds of the causal effect according to equation (4) [35]. Hence, centralising acute stroke leads to a lower bound causal effect on healthcare savings and QoL gain of $US 1581 and 0.01 respectively.

**Table 4:** OLS regression results: healthcare costs (*N* = 1047)

Total costs at 3 months ($US)	Restricted	Full
Centralised	− 1704^a^ (626)	− 1611^b^ (626)
Gender		1255^a^ (522)
Age 25–45 (baseline)		
Age 46–65		2150^a^ (931)
Age 65–96		2317^b^ (661)
IVT received		− 755 (850)
GP (baseline)		
911		−58 (700)
Self-referral		527 (752)
Intra-hospital		509 (1386)
SNIHSS on arrival		762^b^ (139)
Transported by EMS		2847^b^ (661)
Constant	9944^b^ (319)	3838^b^ (808)
R-squared	0.0063	0.1020

*GP* General Practitioner, *sNIHSS* short National Institutes of Health Stroke Scale, *EMS* Emergency Medical Services. Robust standard errors are presented in the parentheses. Inference: ^a^/^b^ indicate significant differences at the 5%/1% level

**Table 5:** OLS regression results: QoL utility values (*N* = 1047)

EQ 5D at 3 months	Restricted	Full
Centralised	0.039 (0.022)	0.018 (0.019)
Gender		−0.029^a^ (0.016)
Age 25–45 (baseline)		
Age 46–65		− 0.027 (0.021)
Age 65–96		−0.126^b^ (0.020)
IVT received		0.048 (0.027)
GP (baseline)		
911		−0.018 (0.022)
Self-referral		−0.009 (0.022)
Intra-hospital		0.004 (0.054)
SNIHSS on arrival		−0.055^b^ (0.003)
Transported by EMS		−0.053^a^ (0.021)
Constant	0.651^b^ (0.011)	0.906^b^ (0.023)
R-squared	0.0033	0.3017

*QoL* Quality of Life, *GP* General Practitioner, *sNIHSS* short National Institutes of Health Stroke Scale, *EMS* Emergency Medical Services. Robust standard errors are presented in the parentheses. Inference: ^a^/^b^ indicate significant differences at the 5%/1% level

### Sensitivity analyses

Deterministic sensitivity analyses were undertaken to test the stability of the observed dominant causal effect of centralising acute stroke care. First, implementing the second validated algorithm to mapp QoL values from the observed mRS scores revealed no change in results (*P* = 0.124). Second, adopting the alternative assumptions underlying the derivation of cost after hospital discharge in the SCM yields healthcare savings of $US 1561 (CI, 524 to 2597) (*P* = 0.003) and $US 1360 (CI, 476 to 2244) (*P* = 0.003), respectively.

## Discussion

In this study we evaluated the causal impact of a centralised stroke care system on healthcare costs and QoL values up to 3 months after hospital discharge, compared to a decentralised stroke care system. To this end we linked the original dataset [9] to the hospital information system comprising patient-level data and used previously published cost estimates [20] and algorithms [21, 22]. We show that centralising IVT lowers costs and increases patients’ health – proving dominance over the decentralised system. On average, the lower bound of the causal impact on healthcare savings was $US 1581, while similarly health outcomes in terms of QoL gain were 0.014 higher. Indeed, studies that did not adequately account for omitted variables bias may have overestimated the effects of centralising IVT, potentially leading to suboptimal budget allocation if adopted by policy makers.

The results are mainly determined by the differences in patient health, as measured with mRS scores, in both stroke care systems. This corroborates our expectation that patients’ health is influenced by the organisation of the healthcare system. Although pre-hospital costs were greater in a centralised system, on average a larger portion of patients in the centralised system become functionally independent again at 3 months (mRS scores 0–1), thereby saving significant healthcare costs by avoiding care in either a nursing home or rehabilitation centre. This is could suggest that higher pre-hospital costs for the centralised system are offset by a decreased length-of-stay in the hospital and avoiding institutional care after hospital discharge due to improved patients’ health. These results suggest that centralising services could contribute to further improving healthcare, as short-term stroke severity is an important predictor of QoL years after the stroke [38]. From a societal perspective it would be interesting to see whether centralisation of acute stroke care would lead to a shift in costs associated with productivity, informal care and additional transport for caregivers. Better outcomes as obtained in the centralised system would have led to higher productivity, and thus added to a more favourable cost difference. Indeed, dominance would have only increased. Furthermore, also the long-term costs incurred for informal care would have been lower in the centralised system, simply as fewer stroke victims would need less of it. Further research is needed to prove these arguments, as data on productivity, informal care and additional transport costs for caregivers are missing in this study.

It is increasingly recognized that stroke care systems centralised at highly specialised tertiary hospital may generate better patient outcomes at lower costs, compared to care offered at community hospitals [12, 39]. Nationally acute stroke care treatment consists of admission to a stroke unit and treatment with IVT, which is currently administered to approximately 15% of the Dutch incident stroke population [9]. Due to an ageing population the number of patients receiving acute treatment is expected to increase substantially in the near future. Expanding services to other hospitals and regions therefore appears to provide great potential for economic as well as patient value. Importantly, costs per patient will likely decrease with large patient volumes due to economies of scale associated with lower training costs of medical specialists and overhead costs for materials and equipment. Additionally, more costly because of economies of scale certainly will apply also in the Netherlands, yet rurality of the Netherlands may be a relative issue. The nearest comprehensive stroke center will hardly ever be further out than say 50 kms. Indeed in a Scandinavian, US or Canadian settings this may be a different issue. In such settings travel time will become a real issue up to a point where certain services simply may no longer be accessible. In acute stroke telemedicine, not taken into account in this study, may become a viable option.

We acknowledge that our study design has some limitations. For example, patient-level data could not be retrieved for actual costs made by patients after hospital discharge. Therefore, we relied on previously published cost estimates in a Dutch setting [20]. We acknowledge this affects the size of the estimate for incremental healthcare costs, but we argue it would not have altered out conclusions, as it has been shown in the literature that healthcare costs increase with functional disability and dependence [3–5, 16–20]. Furthermore, after manipulating the assumptions underlying healthcare use in the deterministic sensitivity analyses, we found that the coefficient only changed moderately. Hence, the dominant effect remained stable. To further understand the effect of centralised stroke care systems on societal costs within clinical practice, future studies may consider following cohorts prospectively from onset to 3 months post-stroke. Furthermore, stroke severity may have been slightly underestimated by using the 5-item short version of the NIHSS. The sNIHSS has been validated for the pre-hospital setting, however the subset of impairments scored is still lower compared to the full version of the NIHSS potentially leading to loss of information on stroke severity. However, this will marginally affect our results, as the sNIHSS is only included as control variable.

Since the results suggest centralising IVT is both cost saving and yields better health outcomes, we dare conclude dominance in terms of cost-effectiveness. We acknowledge that a full cost-utility analysis requires to adopt the Consolidated Health Economic Evaluation Reporting Standards (CHEERS) [40]. This is not feasible as important components are missing in the dataset. For example, extrapolating the results over patients’ lifetime would introduce too much uncertainty, as we would have to rely on transition rates from the literature since follow-up data within applicable cycle-lengths is missing. The latter, however, would not alter the outcome of dominance as after initial treatment failure or success the long-term prognosis is more or less determined, i.e., a higher initial success rate implies both lower long-term costs as well as health benefits [38].

## Conclusions

From this study we conclude that a centralised system for acute stroke care lowers healthcare costs and improves health outcomes within clinical practice. The results are highly relevant for policy makers, as this is the first study to address the issues of selection and unobserved heterogeneity in the evaluation of centralising acute stroke care, hence presenting causal estimates for budget decisions.

## Data Availability

The data that support the findings of this study are available from the UMCG but restrictions apply to the availability of these data, which were used under license for the current study, and so are not publicly available. Data are however available from the authors upon reasonable request and with permission of UMCG.

## Appendix

**Table 6:** stroke incidence per hospital^a^

	All stroke patients	Ischemic stroke patients
Centralized system		
University Medical Center, Groningen	429	361
Ommelander hospital group, Groningen^b^	136	100
Refaja hospital, Stadskanaal	123	112
Decentralized system		
Martini hospital, Groningen	380	332
Medical Center, Leeuwarden	527	469
Hospital the Tjongerschans, Heerenveen	237	203
Hospital Nij Smellinghe, Drachten	265	223
Antonius hospital, Sneek	230	203
Treant care group, location Scheper, Emmen^c^	319	273
Wilhelmina hospital, Assen	266	216
Diaconessenhuis hospital, Meppel	224	202
Hospital Bethesda, Hoogeveen	83	70

^a^Number were taken from an online data repository of the National Health Care Institute (https://www.zorginstituutnederland.nl/) containing data from October 2014 till September 2015

^b^The Ommelander care group, Groningen consists of two community hospital both part of the central system

^c^Treant care group, location Scheper, Emmen did not participate in the observational study performed in 2010 (9)

**Table 7:** Comparison of mean differences in raw and matched data

	Raw	Matched
Age in years	-0.1978049^a^	-0.0796457
Male	0.1285413^a^	0.0057671
IVT received	-0.2216757^b^	-0.058478
sNIHSS on arrival	0.1092078	0.0387365
Mode of referral	0.2681143^a^	0.0242103
Transported by EMS (%)	0.3942428^b^	0.025803

*IVT* Intravenous Thrombolysis, *sNIHSS* short National Institutes of Health Stroke Scale, *EMS* Emergency Medical Services, *km* kilometer. Inference: ^a^/^b^ indicate significant differences at the 5%/1% level based on the mean differences of the two systems

## Abbreviations

ATE: Average Treatment Effect
CHEERS: Consolidated Health Economic Evaluation Reporting Standards
CI: Confidence Intervals
EMS: Emergency Medical Services
EVT: Endo-Vascular Treatment
GP: General Practitioners
IVT: Intra-Venous Thrombolysis
mRS: modified Rankin Scale
OLS: Ordinary Least Squares
PPP: Purchasing Power Parity
QALYs: Qualtiy-Adjusted Life Years
QoL: Quality of Life
SCM: Synthetic Control Methods
sNIHSS: short National Institutes of Health Stroke Scale
UMCG: University Medical Centre Groningen
